# Black Esophagus in a Critically Ill Patient With Septic Shock: A Case Report of a Rare Entity

**DOI:** 10.7759/cureus.91231

**Published:** 2025-08-29

**Authors:** Yoseph M Habte, Binyam M Habte, Esimael M Abdu, Makida M Habte, Shimelis A Yimer

**Affiliations:** 1 Department of Medicine, Ethio Tebib Hospital, Addis Ababa, ETH; 2 Department of Medicine, University of Gondar, Gondar, ETH; 3 Department of Surgery, Teklehaimanot General Hospital, Addis Ababa, ETH; 4 Department of Medicine, Bethel Medical College, Addis Ababa, ETH; 5 Department of Pathology, Ethio Tebib Hospital, Addis Ababa, ETH

**Keywords:** acute esophageal necrosis, black esophagus, case report, critical illness, septic shock

## Abstract

Acute esophageal necrosis (AEN), also known as “black esophagus,” is a rare and severe form of esophageal injury characterized by circumferential black discoloration of the distal esophageal mucosa with a sharp demarcation at the gastroesophageal junction. It commonly presents in critically ill patients with upper gastrointestinal (GI) bleeding, hemodynamic instability, and multiple comorbidities. We present a case of a 65-year-old male with septic shock and multiorgan dysfunction, who was confirmed to have AEN and acute duodenitis on endoscopy and biopsy. The patient was managed conservatively with supportive care, including hemodynamic stabilization, proton pump inhibitors (PPIs), broad-spectrum antibiotics, total parenteral nutrition, and dialysis. Over time, his condition improved, and he was discharged in stable condition. This report emphasizes the need for clinicians to keep AEN in mind when encountering critically ill patients with hemodynamic instability to facilitate prompt diagnosis and effective treatment.

## Introduction

Acute esophageal necrosis (AEN), also known as “black esophagus”, is a rare but severe form of esophageal injury, with an incidence between 0.01% and 0.28%. It is identified endoscopically by a circumferential black discoloration of the mucosa, typically affecting the distal esophagus with a sharp demarcation at the gastroesophageal junction [1,2]. In patients presenting with upper gastrointestinal (GI) bleeding, multiple comorbidities, and hemodynamic instability, this condition should be considered as a potential diagnosis [1]. Given its high mortality rate of 30-50%, early and aggressive systemic resuscitation, along with stabilization and management of underlying medical conditions, is crucial for improving patient outcomes [1,3]. We describe the case of a 65-year-old male admitted to our ICU in shock, in whom endoscopic evaluation and biopsy confirmed AEN along with acute duodenitis.

## Case presentation

A 65-year-old male with no significant past medical history presented with a one-week history of progressive illness. The patient had initially been well but subsequently developed a fever and a dry cough. He had self-administered oral azithromycin for three days without improvement. This had been followed by crampy abdominal pain, diarrhea, jaundice, and acute confusion. He had been admitted to a local hospital with a provisional diagnosis of fulminant hepatitis versus severe acute cholangitis, complicated by septic shock of chest focus and acute kidney injury. Despite broad-spectrum antibiotics, vasopressors, and supportive measures, his condition had remained critical, prompting his transfer to our hospital's ICU.

On arrival, his BP was 100/70 mmHg on noradrenaline weaning; he was tachycardic and hypoxic on high-flow oxygen. Examination revealed icteric sclera, right upper quadrant tenderness, and altered mentation without focal neurological deficits. Laboratory investigations showed leukocytosis (WBC: 24.6 x 10^3^/µL) with neutrophil predominance (94.7%), anemia (Hemoglobin: 9.5 g/dL), creatinine: 6.8 mg/dL, urea: 220 mg/dL, markedly elevated liver transaminases (AST: 582 U/L, ALT: 308 U/L), hyperbilirubinemia (total: 5.1 mg/dL), hypoalbuminemia, coagulopathy (INR: 1.85), and high anion gap metabolic acidosis (Table 1).

**Table 1:** Laboratory investigation results on admission and on discharge

Laboratory Investigations	On Admission (Day 1)	On Discharge (Day 14)	Normal Value
Metabolic Panel			
Creatinine	6.8 mg/dl	2.9 mg/dl	0.67 — 1.17 mg/dl
Urea	220 mg/dl	90.08 mg/dl	17 — 43 mg/dl
Na+	156 mmol/l	139.8 mmol/l	136 — 145 mmol/l
K+	4.9 mmol/l	3.91 mmol/l	3.5 — 5.1 mmol/l
Cl-	114 mmol/l	101.8 mmol/l	98 — 111 mmol/l
Aspartate Transaminase	582.1 U/L	42.42 U/L	2 — 50 U/L
Alanine Transaminase	308.3 U/L	20.65 U/L	1 — 50 U/L
Alkaline Phosphatase	157.8 U/L	261.17 U/L	80 — 300 U/L
Albumin	1.53 g/dL	3.39 g/dL	3.5 — 5.2 g/dL
Total Protein	4.85 g/dL	6.37 g/dL	6.6 — 8.3 g/dL
Bilirubin Total	5.10 mg/dL	1.3 mg/dL	0.3 — 1.2 mg/dL
Bilirubin Direct	3.32 mg/dL	0.3 mg/dL	0.0 — 0.2 mg/dL
Coagulation Profile			
Prothrombin Time	19.8 seconds	13.9 seconds	10.7 — 14.3 seconds
International Normalized Ratio	1.85	1.19	0.8 — 1.2
Activated Partial Thromboplastin Time	38.8 seconds	29.1 seconds	21 — 35 seconds
Arterial Blood Gas Analysis			
PH	7.087 mmHg	7.380 mmHg	7.350 — 7.450 mmHg
PCO_2_	15.4 mmHg	40 mmHg	35 — 48 mmHg
PO_2_	97.4 mmHg	95 mmHg	83 — 108 mmHg
SO_2_	92.5%	97%	95 — 99%
Lactate	1.8 mmol/l	0.8 mmol/l	0.5 — 1.6 mmol/l
Serology			
Hepatitis B Surface Antigen	Negative		
Hepatitis C Virus Antibody	Negative		
Rapid HIV Test	Negative		
C-reactive Protein	133.3 mg/l	5.9 mg/l	0.0 — 5.0 mg/l
Complete Blood Count			
White Blood Cells	24.6 x 10^3^/µL	5.3 x 10^3^/µL	4.0 — 11.0 x 10^3^/µL
Hemoglobin	9.5 g/dl	12.1 g/dl	13.5 — 17.5 g/dl
Platelet	241 x 10^3^/µL	176 x 10^3^/µL	150 — 450 x 10^3^/µL
Lymphocyte Percentage	3.0%	17.4%	15 — 50%
Neutrophil Percentage	94.7%	69.2%	45 — 80%

**Figure 1:**
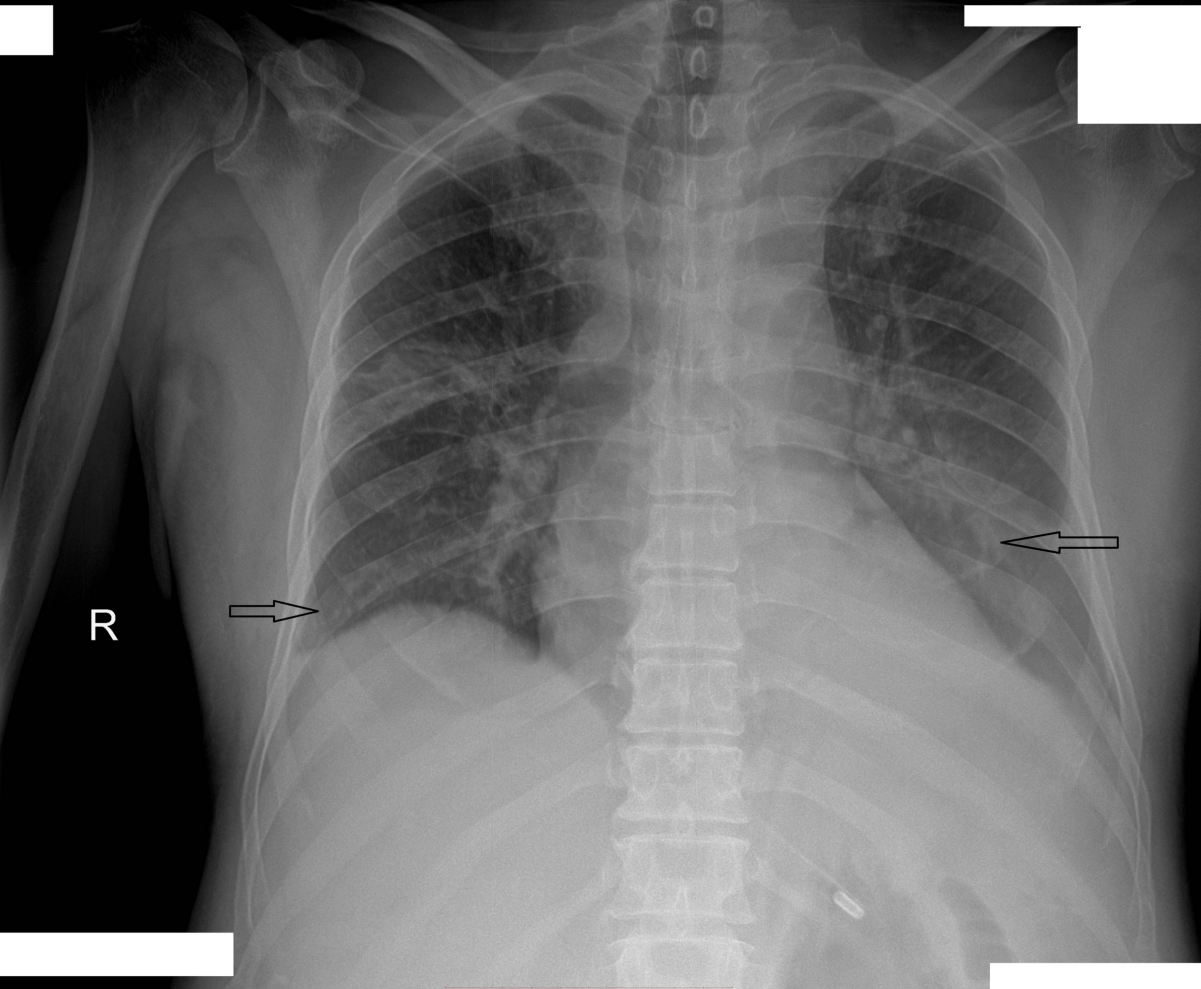
Chest X-ray revealing bilateral lower lobe consolidation

Chest X-ray demonstrated bilateral lower lobe consolidation (Figure 1). Additionally, an abdominal ultrasound showed moderate ascites and cholelithiasis.

**Figure 2:**
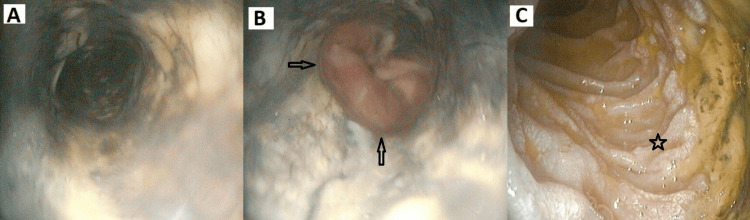
Endoscopic findings (A) Circumferential black discoloration involving the distal two-thirds of the esophagus. (B) Sharp demarcation at the gastroesophageal junction. (C) Ulcerated lesion in the second part of the duodenum consistent with acute duodenitis

The patient had acute respiratory distress syndrome, sepsis-associated acute kidney injury requiring hemodialysis, and atrial fibrillation with rapid ventricular response, controlled with amiodarone. Given his obstructive jaundice, severe sepsis, cholelithiasis, and suspected ascending cholangitis, endoscopic retrograde cholangiopancreatography (ERCP) was performed. During the procedure, circumferential black discoloration involving the distal two-thirds of the esophagus with a sharp demarcation at the gastroesophageal junction was revealed. The stomach mucosa was normal. A large ulcerated lesion was also observed in the second portion of the duodenum, suggestive of acute duodenitis. The papilla was not identifiable, and biliary cannulation was unsuccessful despite several attempts, and further cannulation attempt was limited by unstable cardiorespiratory parameters. Subsequent esophagogastroduodenoscopy with biopsy confirmed these findings (Figure 2).

**Figure 3:**
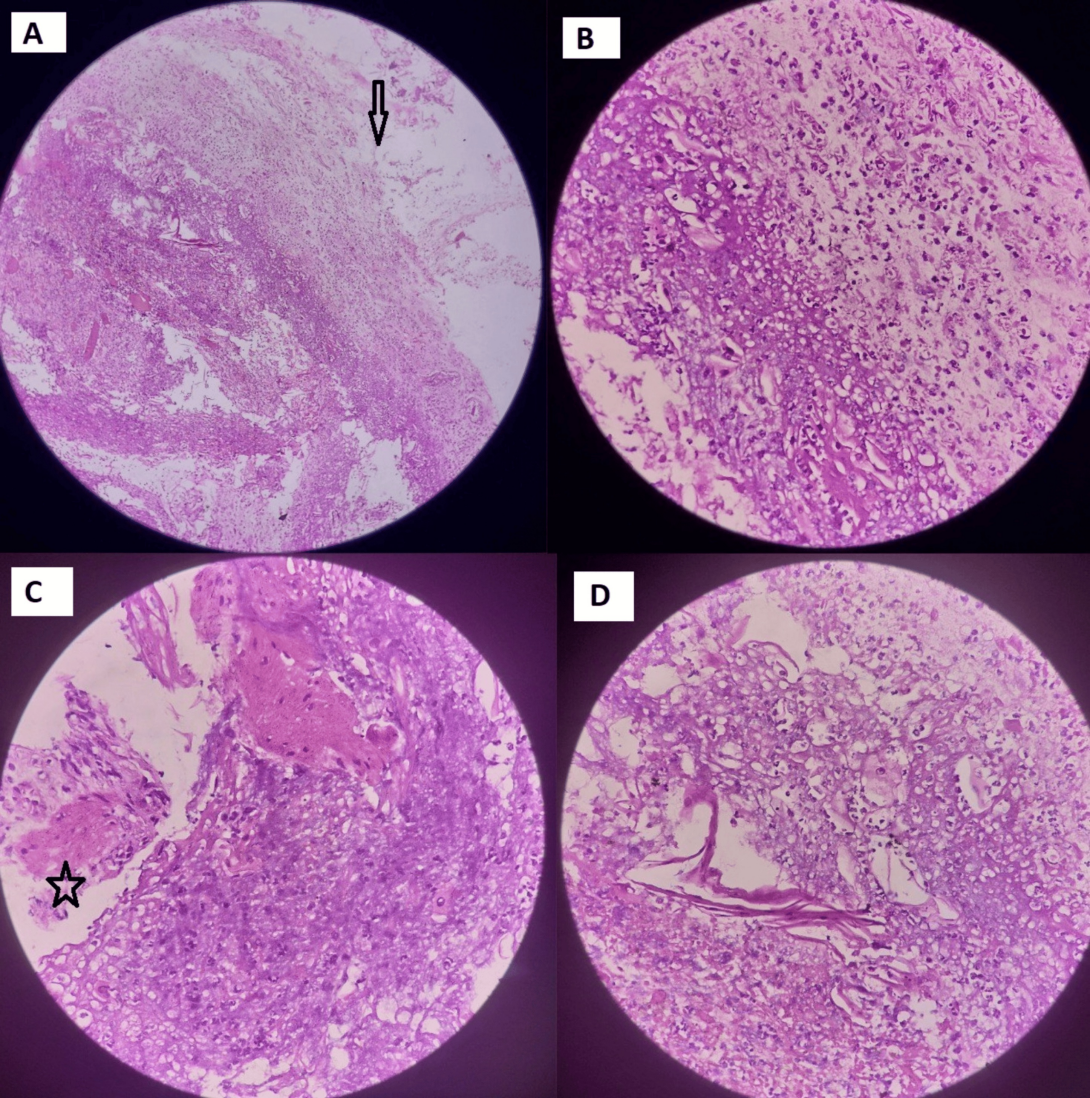
Histopathology from the esophageal biopsy (A) Necrotic mucosa with totally denuded epithelial lining. (B) Neutrophilic infiltration. (C and D) Disrupted muscularis mucosae with fragmented smooth muscle and neutrophilic infiltrates

Histopathological examination of the esophageal biopsy revealed edematous and necrotic tissue infiltrated by neutrophils and macrophages, with loss of the mucosal lining. A fragmented and necrotic muscularis layer was also noted, consistent with acute esophageal necrosis. Duodenal biopsy showed focal erosion with degenerating epithelial cells and neutrophilic infiltrates. The lamina propria was edematous and infiltrated by lymphocytes and eosinophils, consistent with acute duodenitis (Figure 3).

The patient was managed conservatively with strict nil per os (NPO) status, high-dose proton pump inhibitor (PPI) therapy, total parenteral nutrition, intravenous antibiotics (meropenem 500 mg every 24 hours and vancomycin 1800 mg as a loading dose, followed by 900 mg every 48 hours), hemodialysis, and meticulous hemodynamic support. Corticosteroids were discontinued after stabilization. Nasogastric tube placement was avoided to minimize perforation risk.

Over the subsequent days, his mental status improved, renal function showed partial recovery, serum electrolytes returned to normal, inflammatory markers decreased, and other laboratory parameters improved. He was transferred out of the ICU in stable condition for ongoing nutritional and supportive care, and was eventually discharged from the hospital on the 14th day of admission in improved condition with a follow-up appointment.

## Discussion

AEN, often called “black esophagus,” is a rare yet serious condition marked by widespread circumferential black discoloration of the esophageal mucosa, most commonly affecting the distal third. Although its incidence is estimated between 0.01% and 0.28% in large endoscopic studies, post-mortem examinations indicate that it may be underrecognized in critically ill individuals [1,2]. The underlying mechanisms of AEN are thought to be multifactorial, involving ischemia resulting from systemic hypoperfusion, chemical damage due to extensive gastric acid reflux, and compromised mucosal healing capacity as a result of systemic illness or coexisting medical conditions [1,2,4]. Commonly associated conditions include various forms of shock (septic, cardiogenic, or hypovolemic), sepsis, diabetic ketoacidosis, older age, chronic kidney disease, malnutrition, and liver dysfunction [1,4,5]. In line with this understanding, our patient developed esophageal necrosis following an episode of septic shock with multi-organ injury.

Clinically, AEN often presents with upper GI bleeding, such as hematemesis or melena, along with systemic manifestations like hypotension or shock [5]. Complications such as perforation, stenosis, and peristaltic abnormalities may develop and should be carefully assessed when suspected [4,5]. On endoscopy, the hallmark feature is a circumferential black discoloration of the distal esophageal mucosa with a clear transition at the gastroesophageal junction, while the gastric lining typically remains unaffected [1,6]. These typical gross features are sufficient to make a diagnosis without biopsy [2,6], but histology demonstrates necrotic debris, loss of viable epithelium, and necrosis involving tissues at varying depths [2]. In our case, the combination of obstructive jaundice, septic shock with acute kidney injury, and additional comorbidities prompted a timely ERCP, during which the black esophagus was incidentally identified. Subsequently, esophagogastroduodenoscopy confirmed the diagnosis, and microscopic evaluation of biopsy specimens revealed typical histological features described above.

The management of AEN is primarily supportive. Stabilizing the patient’s hemodynamics through fluid resuscitation and addressing the underlying triggers is essential. Intravenous PPIs are routinely administered to suppress gastric acid and prevent further mucosal injury [1,3-5]. Nutritional support must be handled cautiously, typically beginning with bowel rest and parenteral nutrition to avoid additional trauma to the esophagus [4]. Surgical intervention is generally reserved for complicated cases, such as esophageal perforation leading to mediastinitis or abscess formation [1,4,7]. In line with these management principles, our treatment approach included keeping the patient NPO, administering high-dose intravenous PPIs, initiating total parenteral nutrition, providing broad-spectrum antibiotics, and aggressively managing hemodynamic instability.

Although reported mortality rates range from 30-50%, deaths in these patients usually occur due to the underlying condition rather than AEN itself [1,3,8], and the mortality rate directly attributable to AEN is approximately 6% [2]. In our case, the patient experienced a favorable outcome with progressive clinical and laboratory improvement. The patient was transferred out of the ICU and discharged on day 14 in stable condition with outpatient follow-up arranged.

## Conclusions

AEN is a rare but potentially life-threatening condition that should be considered in critically ill patients with upper GI bleeding, hemodynamic instability, and multiple comorbidities. Early endoscopic diagnosis, followed by timely initiation of supportive care, including hemodynamic stabilization, acid suppression, cautious nutritional support, and management of underlying causes, is essential for improving patient outcomes. Although the overall prognosis remains poor due to the severity of associated systemic illness, appropriate and timely intervention can lead to recovery, as demonstrated in our patient.
